# Mental rotation of feet in individuals with Body Integrity Identity Disorder, lower-limb amputees, and normally-limbed controls

**DOI:** 10.1371/journal.pone.0221105

**Published:** 2019-08-16

**Authors:** Kayla D. Stone, H. Chris Dijkerman, Robin Bekrater-Bodmann, Anouk Keizer

**Affiliations:** 1 Department of Experimental Psychology, Helmholtz Institute, Utrecht University, Utrecht, The Netherlands; 2 Department of Cognitive and Clinical Neuroscience, Central Institute of Mental Health, Medical Faculty Mannheim, Heidelberg University, Mannheim, Germany; Universita degli Studi di Udine, ITALY

## Abstract

Body Integrity Identity Disorder (BIID) is a non-psychotic condition wherein individuals desire amputation or paralysis of one or more healthy, fully-functioning limbs (predominantly the legs). Individuals with BIID have been suggested to have a mismatch between the perceived mental representation of the body and its actual physical structure, such that their desired identity matches that of a lower-limb amputee. Accordingly, studies have reported an altered central network involving body representation of the legs in BIID, but its relationship to behavior remains unclear. In the present study, we investigated the integrity of body representation in individuals with BIID, acquired lower-limb amputees, and normally-limbed controls using an online mental rotation task. Participants judged the laterality of left and right foot images presented from different views, orientations, and of different types. We expected BIID participants to be slower for mentally rotating images that corresponded to their affected legs than lower-limb amputees and normally-limbed participants. We found that the groups did not significantly differ in their performance. All participants were slower at judging feet presented in awkward postures than natural postures, replicating previous studies and validating our online paradigm. The results are discussed in terms of the robust nature of visual and sensorimotor lower-limb representations, whether related to the self or as prototype, in the context of disturbed lower-limb integrity.

## Introduction

Body Integrity Identity Disorder (BIID) is a rare condition characterized by a strong and persistent desire to amputate or paralyze one or more healthy limbs [1]. The desire to amputate or paralyze a body part presumably arises from experiencing a mismatch between the perceived mental representation of the body and the actual physical structure and/or boundaries of the body itself [2]. Individuals with this condition, particularly those who desire amputation of a limb, describe themselves as “overcomplete” and as though the limb does not belong to them, experiencing a sense of (non-delusional) disownership over the body part. While research is scant on this subject, it has been growing in recent years [3–7]. The condition manifests before adolescence, usually affects males, and the desire to amputate/paralyze is usually directed towards the lower limbs [2,8]. BIID is not yet included in the most recent version of the Diagnostic and Statistical Manual of Mental Disorders (DSM-V); however, it is set to be included in the next version of the International Classification of Diseases (ICD-11) as Body Integrity Dysphoria [9] (under the category ‘disorders of bodily distress and bodily experience’). At this point, treatment for the condition has not yet been developed.

While BIID is not a product of any apparent brain damage (e.g. [10]), recent imaging studies suggest that there are structural and functional alterations to brain areas that contribute to maintaining a coherent representation of the body (particularly within the sensorimotor system). These areas generally refer to frontal, parietal, and insular areas, but have extended to the thalamus, basal ganglia, and cerebellum as well [3,7,10–12]. For instance, the premotor cortex (PMC) plays a critical role in integrating multisensory information about the body (e.g. [13,14]) and particularly for creating a feeling of body ownership (i.e., the feeling that my body (part) belongs to me; [15,16]). In a sample of 5 BIID participants with unilateral leg amputation desire, Van Dijk et al. [10] revealed that the ventral PMC shows reduced activity in response to tactile stimulation of the affected limb in comparison to the unaffected limb. Blom et al. [3] revealed reduced grey matter volume in the left PMC in a sample of 8 individuals with BIID. Parietal areas, such as the superior parietal lobule (SPL) and the inferior parietal lobule (IPL), which play a critical role for body awareness [17,18], processing configuration of the body [19], and the overall representation of the body [20], are also affected in BIID. Specifically, McGeoch et al. [12] showed, in a sample of 4 BIID participants, that activity of the SPL was reduced in response to tactile stimulation on the affected compared to unaffected limb. In line with this, Hilti et al. [4] revealed reduced cortical thickness of the right SPL and reduced cortical surface area of the secondary somatosensory cortex and anterior insula (involved in overall awareness of the bodily state [21]) in a sample of 13 participants with amputation-variant (i.e. those who desire amputation of a limb) BIID. Moreover, the primary somatosensory cortex (SI), which holds a somatosensory map of the body, showed reduced cortical surface area, *particularly* for the leg/foot representation in this sample [4]. The same individuals (as in [4]) also had structural and functional hyperconnectivity in their sensorimotor areas [7]. Also, subcortical areas like the thalamus and basal ganglia [11] and the cerebellum [3] have displayed anatomical alterations in BIID participants. In addition, a recent study showed that brain activity while viewing images of oneself that were edited in a way that the participant appeared to be amputated could predict whether the participant belonged to the amputation-variant BIID (or specifically: xenomelia, meaning ‘foreign limb’) or control group. These predictive brain regions (like the IPL, SPL, caudate nucleus, occipital areas) overlapped with the areas responsible for creating a coherent representation of the body [5]. However, it remains open whether these brain alterations represent a consequence or a cause of BIID [22].

The growing evidence for an altered or perhaps even impaired representation of the body, particularly within the sensorimotor system, in individuals with BIID provides support for the claims that these individuals internally identify as an amputee. This is highlighted in the following statements by participants in our present online investigations:

“My body doesn't react differently when it comes to my BIID-body part but there is a very distinct feeling below a certain place where it feels like an attachment that isn't supposed to be there. Actually, a bit like a prosthetic [limb]—it's there to keep me up and walking but it is an attachment.”

“My brain tells me that I am a left below knee amputee and my leg should end 5 inches below that knee.”

“For me, BIID is best described as my body feeling like two distinct halves, one of which isn't really on the right 'wavelength'; legs are attached and fully functional, but for some inexplicable reason, I am aware they shouldn’t be.”

In addition, BIID has several parallels with somatoparaphrenia [23], a condition that manifests after stroke, usually following damage to the right hemisphere [24]. In both BIID and somatoparaphrenia, individuals feel like a body part does not belong to them. However, in contrast to somatoparaphrenia, in BIID, the feeling of disownership is not delusional and is not the product of any apparent brain damage. The similarities between BIID and somatoparaphrenia have been explored theoretically [23] and experimentally [25] before. For instance, Romano et al. [25] revealed that, like patients with somatoparaphrenia [26], people with BIID show a reduced anticipatory skin conductance response to stimuli approaching the disowned (to-be-removed) limb. The authors suggest that this altered physiological response could be due to a poorly inscribed central representation of the unwanted limb [25].

Though, few studies have examined the behavioural outcome of these altered (or poorly established) body representations. Recently, Macauda and colleagues [6] explored the implicit (via an implicit association task) and explicit (via a questionnaire) attitudes about amputated and non-amputated bodies in a sample of BIID participants, lower-limb amputees, and normally-limbed control participants. They showed that BIID participants have a stronger implicit and explicit preference for amputated bodies, when compared to amputees and normally-limbed controls. The authors suggest that this implicit preference for amputated bodies in BIID “might rely on a stored body model independent of the physical body”, e.g. an internal body model formed *without* one of the legs. In addition, Lenggenhager and colleagues [27] showed that BIID individuals with unilateral lower-limb amputation desire experience a more vivid rubber foot illusion (i.e. an illusion of ownership over an artificial foot during congruent visuotactile stimulation of one’s own and the artificial foot) for the affected foot compared to control participants, perhaps reflecting a poorly established central lower-limb representation. Thus, this finding also suggests that the integrity of the internal model of the affected limb in BIID is integral in mediating the multisensory experience of the body. For example, it has been shown that individuals who deny ownership over the body or its parts as a result of tumor or stroke show increased susceptibility to the rubber hand illusion [28,29]. Thus, disruptions to these internal body models seem to affect multisensory processes involving the affected limb.

Taken together, these findings corroborate the quoted statements above and provide insight how the underlying representations of the body can affect behaviour. However, an understanding of body representation(s) in BIID is still in its infancy. How can we further behaviourally examine this experienced representation of the body, and more specifically the integrity (or perhaps lack thereof) of the affected limb in BIID? As we still do not know the origins of the disorder, it could prove beneficial to explore different forms of body representation in these individuals in order to elucidate the underlying mechanisms.

While there are several ways to investigate body representation, tasks involving mental rotation of body parts are a simple and elegant way to investigate the integrity of body representations in the brain. In a standard version of the task, participants are asked to make judgements about the laterality of a pictured body part [30]. The left or right body part can be displayed in different orientations (from first-person perspective to third person perspective) and from different views (top or bottom of the body part). In order to successfully make a judgement about the body part’s laterality, participants will internally compare the displayed image to their own body part, and then mentally rotate their body part until it matches the posture of the pictured part, making an imagined spatial transformation of the limb [30] which is reflected by prolonged reaction times in the laterality judgment. Evidence for this egocentric mental rotation strategy during the task comes from behavioural and imaging data. For example, participants are slower and less accurate when making judgements about body parts viewed from a third-person perspective compared to a first-person perspective or from the bottom of the body part compared to the top [31–34]. And while the same pattern of behaviour also emerges for mental rotation of objects or letters, it is exaggerated for body parts [35,36], reinforcing the suggestion that reference to one’s own body representation is critically involved in the process. The sensorimotor system, including bilateral PMC, SPL, the intraparietal sulcus, left IPL, left insula, primary motor area, and SI, plays an active role in mental rotation (e.g. [19,33,37–41], for a review see [42]). Specifically, when compared to mentally rotating objects, mental rotation of body parts engages additional sensorimotor areas implicated in preparing a movement (primary motor cortex, SPL, IPL, insula, primary visual cortex, and other frontal areas, see [43]). This provides further support that an intact sensorimotor representation of the body is important for mental rotation of body parts. Interestingly, several of the brain areas involved in mental rotation tasks overlap with the ones that are altered in BIID (e.g. SPL [12], IPL [4], PMC [2], SI [4]).

Several studies have shown that performance during mental rotation of body parts is modulated by central and peripheral representations of the body. For instance, individuals with an acquired upper-limb amputation are slower and less accurate at making judgements about the laterality of the amputated hand, particularly if the dominant hand was amputated, when compared to the intact hand and to control participants [34]. With respect to the lower limbs, only two studies have investigated mental rotation of the missing body part [44,45]. In both studies, having an amputation did not lead to clear deficits in performance. For instance, Boccia et al. [45] showed that while lower-limb amputees performed similar to controls (at a behavioural level), lower-limb amputees showed differences in brain activity that were specific to the body representation network during the task. In another study, Curtze et al. [44] revealed that while normally-limbed controls showed an advantage for making judgements about the right feet (presumably because of foot dominance), lower-limb amputees did not show a laterality effect. Moreover, the presence/absence of phantom sensations during the experiment influenced reaction times (though this interaction was not explicitly described). These findings encourage further exploration of the effects of lower-limb amputation on mental rotation. Performance regarding mental rotation of body parts is also affected in individuals who are paralyzed ([46], c.f. [47,48]), suffer from long-term pain [49,50], or have movement disorders that elicit long-term awkward and fixed postures (such as in dystonia [51,52]), emphasizing the important role of peripheral input during mental rotation of body parts. It is important to note that the impairments in performance (delayed reaction times, increased error rates) are usually specific to the affected body part. The effects of posture of the own body part on mental rotation reflects this too [31,33,51,53]. For instance, placing the hands behind the back slows reaction times for laterality judgements of hands (but not for judgements of feet), but only in right-handed participants [31]. However, it remains unknown whether foot posture during mental rotation of feet affects laterality judgements. Finally, other aspects of bodily awareness like the feeling of ownership over one’s body parts might also disturb mental rotation. Van Stralen et al. [54] recently showed that stroke patients with self-reported body ownership impairments displayed deficits in making left-right orientation judgements about bodies. These studies highlight the importance of an intact body representation (including an intact sense of ownership) for making mental transformations of the body. Individuals can use visual and sensorimotor cues about the body part in order to solve the task, but also remembered structures of a prototypical body. Therefore, the task likely taps into several aspects of body representation, including sensorimotor (i.e., the body schema [55]) and structural (i.e., the body structural description [44]) representations. Which aspects of body representation are impaired in BIID, however, remain unknown.

Therefore, in the current study, we ask the question: is mental rotation of the feet impaired in people with lower-limb BIID? As BIID is a rare condition and obtaining large sample sizes in a fixed lab setting is a challenge (for reference, see samples sizes in the studies mentioned above), we took an online study approach to explore this question. We tested 16 participants with BIID, 19 individuals with an acquired lower-limb amputation, and 33 age- and sex-matched normally-limbed controls on a body part mental rotation task using the online platform Gorilla (www.gorilla.sc/about). Participants were asked to make laterality judgements about images of feet presented on-screen. Reaction times and overall error rates were examined. Both normally-limbed individuals and lower-limb amputees have a normal innate representation of their legs. However, in the lower-limb amputees, the leg itself has been physically compromised. However, the physical absence of a foot does not seem to influence mental rotation abilities [44]. In contrast, BIID participants seem to have a disturbed innate representation of their leg(s), even though they are physically intact. Therefore, we hypothesized that participants with BIID would be slower in making judgements about their affected parts (in this case, their feet) when compared to their non-affected body parts and to both amputee (i.e. a clinically-oriented sample) and normally-limbed (i.e. healthy, intact control sample) groups.

## Methods

### Participants

#### Participants with BIID

Sixteen participants (14 males, 1 female, 1 missing response) with self-report unilateral BIID (average age: 46.9 ± 13.8 SD, with missing age data from 2 participants) completed the study. Eight individuals desired right leg amputation and eight individuals desired left leg amputation. On average, participants stated that their desire to have an amputation started around the age of 8, ranging from age 5 to 20 (mean = 8.7, SD = 4.2 years). Most individuals (*n* = 13) had completed higher education or a university degree, while two individuals completed secondary school (one response was missing). The mother tongues of participants were German (*n* = 9), English (*n* = 3), and Dutch (*n* = 1). Responses regarding mother tongue were missing for three participants. Average number of years with BIID was 38.2 (± 15 SD, range 10 to 59). Most (*n* = 11) were self-diagnosed with BIID, while the remaining stated being diagnosed by a psychiatrist. Eight individuals reported a specific trigger at the moment their first BIID feelings started, and in all cases, it involved seeing an amputee at a young age. Participants were recruited through online BIID support group forums (https://groups.yahoo.com/neo/groups/fighting-it/info and https://forum.biid.ch/).

#### Lower-limb amputees

Nineteen individuals with a unilateral acquired lower-limb amputation (14 males, 5 females), with an average age of 49.2 ± 7.2 SD took part. Thirteen individuals had a right leg amputation whereas six individuals had a left leg amputation. The reasons for amputation were accident (*n* = 15), tumor (*n* = 2), consequence of injury (*n* = 1), and infection (*n* = 1). Most individuals (*n* = 10) had completed higher education or a university degree, while six individuals completed secondary school and two completed primary school (1 response was missing). The mother tongues of the participants were German (*n* = 18) and Russian (*n* = 1). Average number of years with an amputation was 25.8 (± 12.7 SD, range 8 to 47). Noteworthy, 8/19 participants were experiencing phantom limb sensations at the time of the task. Moreover, 17/19 participants reported using prostheses on a regular basis (the other two never used prostheses). When asked “how strongly do you feel the prosthesis is part of your body?” on a scale from 0 (prosthesis feels foreign to my body) to 10 (prosthesis feels like part of my body), average score was 5.5 ± 4.0 SD. When asked “how much control do you have over the movements of your prothesis?” on a scale from 0 (no control) to 10 (full control), average score was 7.1 ± 2.3 SD. Participants were recruited via telephone and email from the database described in Bekrater-Bodmann et al. [56]. Participants were asked about their English proficiency prior to participation. English was sufficient for most participants. However, to ensure that they understood the study, we offered to guide participants through the questionnaires (via the telephone). Two participants were guided through the questionnaires.

#### Normally-limbed controls

Thirty-three age- and sex-matched (to the BIID and amputee groups) control participants (26 males, 6 females, 1 preferred not to say) completed the study, with an average age of 43.5 years ± 11.6 SD. Most individuals (*n* = 28) had completed higher education or a university degree, while three individuals completed secondary school (two responses were missing). The mother tongues of the participants were English (*n* = 18), Italian (*n* = 3), German (*n* = 2), Spanish (*n* = 2), Portuguese (*n* = 2), Dutch (*n =* 1), Turkish (*n* = 1), Estonian (*n =* 1), Slovak (*n =* 1), Urdu (*n =* 1), and Swedish (*n =* 1). Participants were asked if they had an amputation or if they ever felt like a part of their body did not belong to them, in which all responded ‘no’. They were recruited using Prolific (https://prolific.ac/).

See supplementary material S1 Table for characteristics of participants. All participants gave digital informed consent (by clicking “OK” on digital consent form) in accordance with the Declaration of Helsinki and the approval of the local ethics committee (protocol number: FETC16-011) before participating in the study.

### Materials and task design

All questionnaires and the mental rotation task were administered in English.

### Questionnaires

#### Demographics and medical history

All participants completed a general questionnaire which included questions about demographics (e.g. age, sex, ethnicity) and medical history (e.g. presence of psychiatric or chronic medical disorder). BIID participants completed a more elaborate version of the questionnaire with additional questions about their BIID (modified version of the BIID Phenomenology Questionnaire [2]). Amputee participants also completed an elaborate version of the general questionnaire with additional questions about the amputation and the presence of phantom limb sensations.

#### Screening for mental illness

We used self-report questionnaires to examine the prevalence and extent of common psychiatric disorders in our sample. We used the **Modified Mini International Neuropsychiatric Interview Screen (MINI screen)** to screen for current mood, anxiety, and psychotic disorders [57,58]. Participants made binary (yes/no) responses to 25 statements. A sum of ≥10 indicates possible psychiatric disorder. To screen for borderline-typical symptomatology, we administered the **Borderline Symptom List (BSL– 23**; [59]). Participants rated their agreement with each of the 23 statements from 0 (not at all) to 4 (very strong) regarding their feeling/experiences in the course of the last week. To obtain a total score, responses are summed and divided by 23 (number of items). This score indicates the severity of borderline personality symptoms [60]. This scale was included as individuals with BIID tend to score higher on inventories assessing borderline symptoms [4,7,11]. However, as noted by previous authors, this trend has likely been driven by questions relating to body dissatisfaction, e.g. “I found my body completely unacceptable in its present state”. However, these previous examinations have included the full borderline symptoms list (95 items). The version with 23 items, used in the current study, contains considerably less questions pertaining to body dissatisfaction but has similar reliability, internal consistency, and high level of sensitivity in comparison to the BSL-95 [59].

#### Functional impairment due to BIID or amputation

The **Sheehan disability Scale (SDS)** [61] was used to assess functional impairment in work/school, social, and family life due to having BIID or due to having an amputation. Control participants did not complete this questionnaire. The scale consists of three statements regarding how much the symptoms have disrupted work/school, social life, and family life, in which participants had to respond on a scale from 0 (not at all) to 10 (extremely). Scores >5 on any of the questions suggest significant functional impairment in that area due to the condition (in this case, amputation (amputees) or desire for amputation (BIID)). In addition, participants indicated how many days were lost and how many days were underproductive in the past week due to the condition.

#### Foot dominance

As hand dominance has been shown to influence laterality judgements during a mental rotation task [62] and since it has been suggested to be similar for foot judgements [44], we assessed foot dominance in our sample. To do so, controls and BIID participants completed the modified version of the **Waterloo Footedness Questionnaire (WFC)** [63], a 13-item questionnaire which assessed foot preference for different scenarios (e.g. for kicking a ball). For ten of the items, participants indicated which foot they preferred for different activities (like kicking a ball) on a scale from -2 (left always), -1 (left usually), 0 (equal), +1 (right usually), +2 (right always). The remaining three items included open-ended questions regarding injury or special training with one foot. The responses are summed and responses <0 suggest left-footedness and >0 suggest right-footedness. Amputees did not complete this questionnaire, but were simply asked “Before the amputation, which leg did you USUALLY prefer to use for activities such as kicking a ball?”.

#### BIID-specific questionnaire

In addition to the modified BIID Phenomenology questionnaire, BIID participants additionally completed the **12-item Zurich Xenomelia Scale (ZXS)** [64], which consists of three subscales pertaining to 1) the strength of the participant’s amputation (or paralysis) desire, 2) the participant’s erotic attraction to amputees/being an amputee, and 3) the extent to which the participant engages in pretending behaviours (i.e. simulated the bodily state of being amputated or paralyzed). Participants could rate their agreement with each statement from 1 (strongly agree) to 6 (strongly disagree). Items 1 (reverse-scored), 2, 5 (reverse-scored), 10 are part of the ‘pure amputation (paralysis) subscale’, items 3, 6 (reverse-scored), 9 (reverse-scored), 12 are part of ‘erotic attraction’ and items 4, 7, 8 (reverse-scored), 11 (reverse-scored) are part of the ‘pretending behavior’ subscale. Our ZXS was modified in such a way that the word ‘amputation’ was replaced with ‘paralysis’ for participants who desire paralysis.

### Mental rotation task

**Fig 1:**
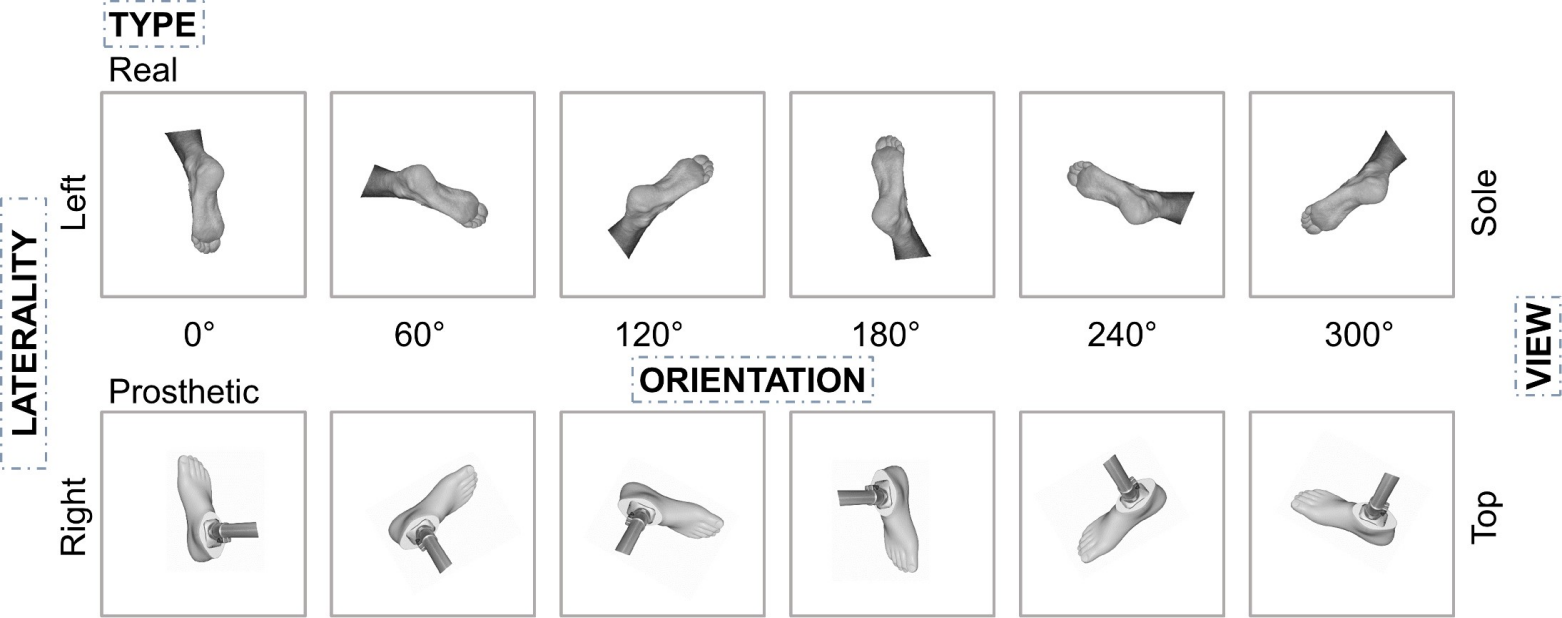
Example of stimuli used in mental rotation task. The image is a visualization of a portion of the stimuli used in the mental rotation task. Note that real feet were also presented from the top view with right laterality in all orientations. Likewise, prosthetic feet were also presented from the sole view with left laterality in all orientations. Use of these stimuli yielded a 6 (orientation) x 2 (side) x 2 (type) x 2 (view) design.

Stimuli consisted of 48 greyscale images (dimensions: 400 x 400 pixels) of feet, which were from part of the set of images used by Curtze et al. [44]. The images included an equal number of left and right feet that could be real or prosthetic, presented from a plantar or dorsal view, in one of six orientations: 0° (first-person perspective), 60°, 120°, 180° (third-person perspective), 240°, or 300°. See Fig 1.

### Procedures

As the experiment was conducted online, participants were free to complete the experiment from a location of their choosing. Order of task and questionnaires was randomized for each participant. All participants completed a series of questionnaires (specific to participant group) and the mental rotation task. As this was part of a larger study, participants also completed an emotional body map task [65] and a modified version of the Toronto Alexithymia Scale. Those data were part of a separate project, but collected together in this study for convenience, and therefore are not reported here.

**Fig 2:**
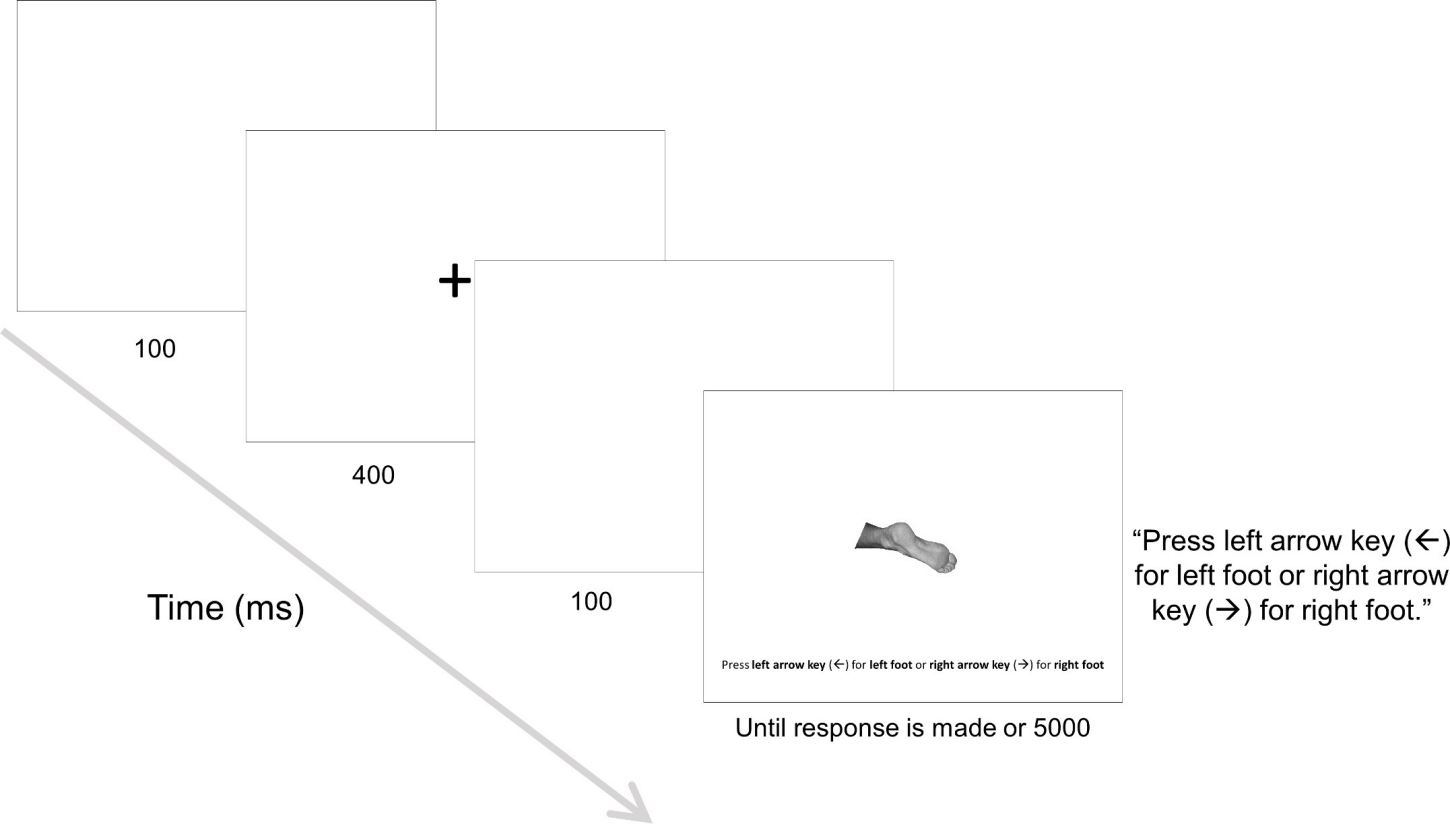
Example trial in the mental rotation task. Image depicts the sequence for a single trial in the mental rotation task. Participants responded to the laterality of the pictured foot by pressing the left arrow key (for left foot judgement) or the right arrow key (for right foot judgement).

For the mental rotation task, participants were instructed to keep their foot or feet flat on the floor and to not change posture of their foot or feet during the experiment. They were instructed to judge, as quickly and accurately as possible, whether a pictured foot shown onscreen was a left foot or a right foot by pressing the left or right arrow key, respectively, on the keyboard. At the start of each trial, a blank white screen appeared for 100ms, followed by a black fixation cross in the center of the screen for 400ms, with a pause (blank screen) of 100ms after the presentation of the cross. Then an image of a foot appeared in the center of the screen. The image remained onscreen until the participant made a response, or if 5000ms had passed, see Fig 2. Participants completed four blocks of trials, consisting of 96 trials each, which would each last approximately four minutes. All blocks had the same stimuli (i.e. presented two times for each possible combination of characteristic, e.g. left prosthetic foot viewed from the sole at 120°), but presentation of stimuli was randomized within each block. Following a block of trials, participants were encouraged to take a break (duration chosen by participant). After the final block, participants were asked if they had changed posture during the experiment and if so, they were prompted to describe this posture change in a comment box (e.g. “I crossed my left foot over my right foot”). Participants completed a total of 384 trials.

Participants had as much time as they wanted to complete the task and questionnaires. After completion, participants were directed to a page with debriefing text.

### Data analysis

Data were processed and analyzed in Microsoft Excel (v. 2016) and MATLAB (v. 2017a). For the mental rotation task, reaction time (RT) referred to the time between stimulus onset and response. In Gorilla™ (www.gorilla.sc), a timestamp is recorded using JavaScript’s “performance.now()” when the stimulus appears and then a second timestamp is taken once a response (key press) is made. The difference between the two timestamps is coded as the reaction time (https://gorilla.sc/support/info/timing). RTs less than 300ms or greater than 4500ms were removed from analysis. While many similar studies choose a stricter criterion, i.e. < 500ms [31,32,38,44,46,66,67], we chose a more liberal range as average RTs for mental rotation of body parts is relatively unknown for the amputee and BIID groups. Median percentage of trials removed for the BIID group was 2.3% (2.0–2.8 interquartile range (IQR)), 2.6% (2.4–5.2 IQR) for the amputee group, and 2.6% (2.3–4.4 IQR) for the normally-limbed control group. The Kruskal-Wallis test on the median percentage was not significant (χ2(2) = 3.8, *p* = 0.145). Only RTs with a correct response (i.e. valid trials) were considered for analysis. The median percentage of removed trials that were < 300ms *and* correct was 0% (0–0 IQR). The median percentage of trials that were >4500ms *and* correct were removed per group as follows: BIID [(0.39% (0–0.5 IQR)], amputees [(0.26% (0–1.3 IQR)], normally-limbed controls [(0.52% (0–1.0 IQR)]. Median reaction times for each stimulus type per participant were log-transformed to correct for the positive skew in the data and to facilitate parametric analysis. For BIID and amputee participants, data were sorted by “affected” and “unaffected” (corresponding to left and right in controls) for homogeneity in analysis. Error rate was calculated by dividing the number of correct valid trials by total valid trials, resulting in a proportion from 0 to 1. Partial eta-squared (η^2^) and Cohen’s *d* were used to show effect sizes. When the assumption of sphericity was violated, the Greenhouse-Geisser correction was applied.

The design was as follows: 6 (orientation) x 2 (laterality) x 2 (type) x 2 (view) x 3 (group). Specifically, following the results of the repeated-measures ANOVAs on RTs, we hypothesized that BIID participants would be slower and less accurate at making judgements about the affected versus unaffected foot (expected: group x side interactions, compared to lower-limb amputees and normally-limbed controls). In the cases where both a main effect and an interaction effect was present, only the highest-level interaction was further explored.

With respect to the questionnaire scores, the Modified MINI was compared between the groups using a one-way ANOVA. As BSL-23 scores were not normally distributed as revealed by Shapiro-Wilk tests (BIID: W = 0.4, *p* < 0.0001, amputees: W = 0.8, *p* = 0.006, controls: W = 0.8, *p* = 0.001), a Kruskal-Wallis test was used to compare groups (with follow-up Mann-Whitney U tests). SDS scores (not normally distributed as revealed by a Shapiro-Wilk test (particularly for the work subscale; BIID: *p* = 0.006; amputees: *p* = 0.02) were compared between amputees and BIID participants using Mann-Whitney U tests. Descriptive results are given about the 12-item ZXS (BIID participants only). Finally, we provide explorative covariance analyses in the supporting material (S1 File), looking at the influence of years since amputation/with BIID, use of prostheses, the presence/absence of phantom limb sensations, and posture change during the study.

## Results

### Questionnaires

#### General demographics of participants

The one-way ANOVA with age as the dependent variable revealed was not significant (*F*(2,30.9) = 2.3, *p* = 0.1). The chi-square test on the distribution of sex across the groups was also not significant (χ^2^(4) = 3.3, *p* = 0.5).

#### Screening for mental illness

**Modified MINI.** The difference between groups on the scores of the Modified MINI was not significant (*F*(2,67) = 0.8, *p* = 0.9, η^2^ = 0.003). The average scores were 4.3 ± 4.0 SD (normally-limbed controls), 4.5 ± 3.6 SD (amputees), and 4.0 ± 3.5 SD (BIID participants). There were two normally-limbed, two amputees, and two BIID participants who had a score ≥10, suggesting a possible mood, anxiety, or psychotic disorder. This was corroborated, for some participants, by their general questionnaire data. Of the two normally-limbed controls with a score ≥10, one reported having bipolar disorder. Of the two amputees, one reported depression with comorbid emotional-unstable personality disorder of the borderline type. Of the two BIID participants, one reported depression. These participants were not excluded from the analyses. The remaining participants with a high Modified MINI score did not report a past or present psychiatric illness.

**BSL-23.** There was a significant difference between groups on the BSL-23, as revealed by a Kruskal-Wallis test (χ^2^(2) = 15.0, *p* = 0.001). Follow-up Mann-Whitney U tests revealed a significant difference between the BIID and amputee groups (*U* = 56, z = -3.1, *p* = 0.001), between the BIID and normally-limbed group (*U* = 92, z = -3.6, *p* < 0.0001), but not the normally-limbed and amputee groups (*U* = 301.5, z = -0.2, *p* = 0.8). That is the BIID group had higher scores (median = 1.3, IQR = 0.8–2.2) on the BSL-23 than the amputee (median = 0.4, IQR = 0.2–0.9) and normally-limbed (median = 0.5, IQR = 0.2–0.9) groups, in line with previous reports showing elevated borderline scores in this population [4,7,11].

#### Functional impairment due to BIID or amputation

**Sheehan Disability Scale (SDS).** Participants with BIID revealed significantly higher levels of impairment due to illness with respect to work (*U* = 80.5, z = -2.3, *p* = 0.01) and family life (*U* = 93.5, z = -1.9, *p* = 0.05) compared to amputees. Scores were similar between the two groups for social life (*U* = 107, z = -1.5, *p* = 0.1). See supplementary S1 Table for scores per participant. However, on averages, scores for all subscales and for both groups were not significantly greater than 5 (*p* ≥ 0.1). For amputees, median number of days lost in the last week due to having an amputation was 0 (0–1 IQR). For BIID participants, median number of days lost in the last week due to having BIID was also 0 (0–1 IQR). Amputees had a median of 0 (0–1 IQR) underproductive days due to the amputation, and participants with BIID had a median of 3.5 (0.75–5.25) underproductive days due to BIID. Normally-limbed controls did not complete this scale. The Mann Whitney-U test between the groups for days lost was not significant (*U* = 136, *p* = 0.78). However, BIID participants reported to have significantly more underproductive days than amputees (*U* = 85, *p* = 0.02).

#### Foot dominance

Thirteen amputees reported right foot dominance before amputation, four reported left dominance and two reported ambidexterity. Eleven individuals with BIID had a positive score on the footedness questionnaire, suggesting a right foot dominance, while three individuals had a negative score, suggesting left foot dominance (mean = 6.6 ± 10.8 SD). One individual with BIID did not complete the questionnaire. Twenty-seven normally-limbed participants had a positive score on the questionnaire, while the remaining six had a negative score (mean = 8.4 ± 6.6 SD). A chi-square test indicated no significant differences in foot dominance across groups (χ^2^(4) = 5.7, *p* = 0.2).

#### BIID-specific questionnaire

**12-item Zurich Xenomelia Scale (ZXS).** Only BIID participants completed this scale. Average scores ± standard deviations for each subscale were as follows: 4.5 ± 0.4 (pure amputation desire), 3.7 ± 0.7 (erotic attraction), and 3.5 ± 0.9 (pretending behaviours). Total average score was 3.9 ± 0.4 out of a possible 6. These scores are in line with previous studies using this scale to describe BIID samples (e.g. [4,11,64]).

### Mental rotation task

#### Reaction times

A 6 (orientation) x 2 (laterality) x 2 (type) x 2 (view) x 3 (group) repeated-measures ANOVA was conducted on the mean of the log-transformed reaction times, with group as the between-subjects factor. There was a main effect of orientation (*F*(3.2, 211.8) = 299.1, *p* < 0.0001, η^2^ = 0.82), indicating that participants were slower to react as the images moved from first- to third-person perspective and back from third- to first-person perspective (e.g. 0 to 180 degrees, from 180 to 300 degrees). There was a main effect of view (*F*(1, 65) = 315.9, *p* < 0.0001, η^2^ = 0.82), indicating that participants were faster to react to feet viewed from the top compared to the sole. There was a main effect of type (*F*(1, 65) = 4.3, *p =* 0.04, η^2^ = 0.06), indicating that participants were faster to react to prosthetic feet than real feet.

There was an interaction between orientation and type (*F*(3.8, 253.2) = 5.5. *p* < 0.0001, η^2^ *=* 0.07). There was also an interaction between orientation and view (*F*(3.4, 221.8) = 53.0, *p* < 0.0001, η^2^ = 0.44). Critically, there was an interaction between orientation, type, and view (*F*(4.4, 290.6) = 537.4. *p* < 0.0001, η^2^ *=* 0.1). To further investigate differences between the levels of this interaction, we first visually explored the data by plotting it. Because the difference between top and sole views were not the same across orientations, we inspected the difference between the views within each level of orientation, separately for both foot types, using paired samples t-tests (critical Bonferroni-corrected *p* = 0.004). There was a difference between top and sole views for real feet at 180 degrees (*t*(67) = -5.8, *p* < 0.001, and *t* ≥ 5.9 *p* < 0.001 for top versus sole in all other orientations), there was no significant difference between reaction times for top and sole views of prosthetic feet oriented at 180 degrees (*t*(67) = -0.6, *p* = 0.5, but *t* ≥ 8.3 *p* < 0.001 for all other orientations).

**Fig 3:**
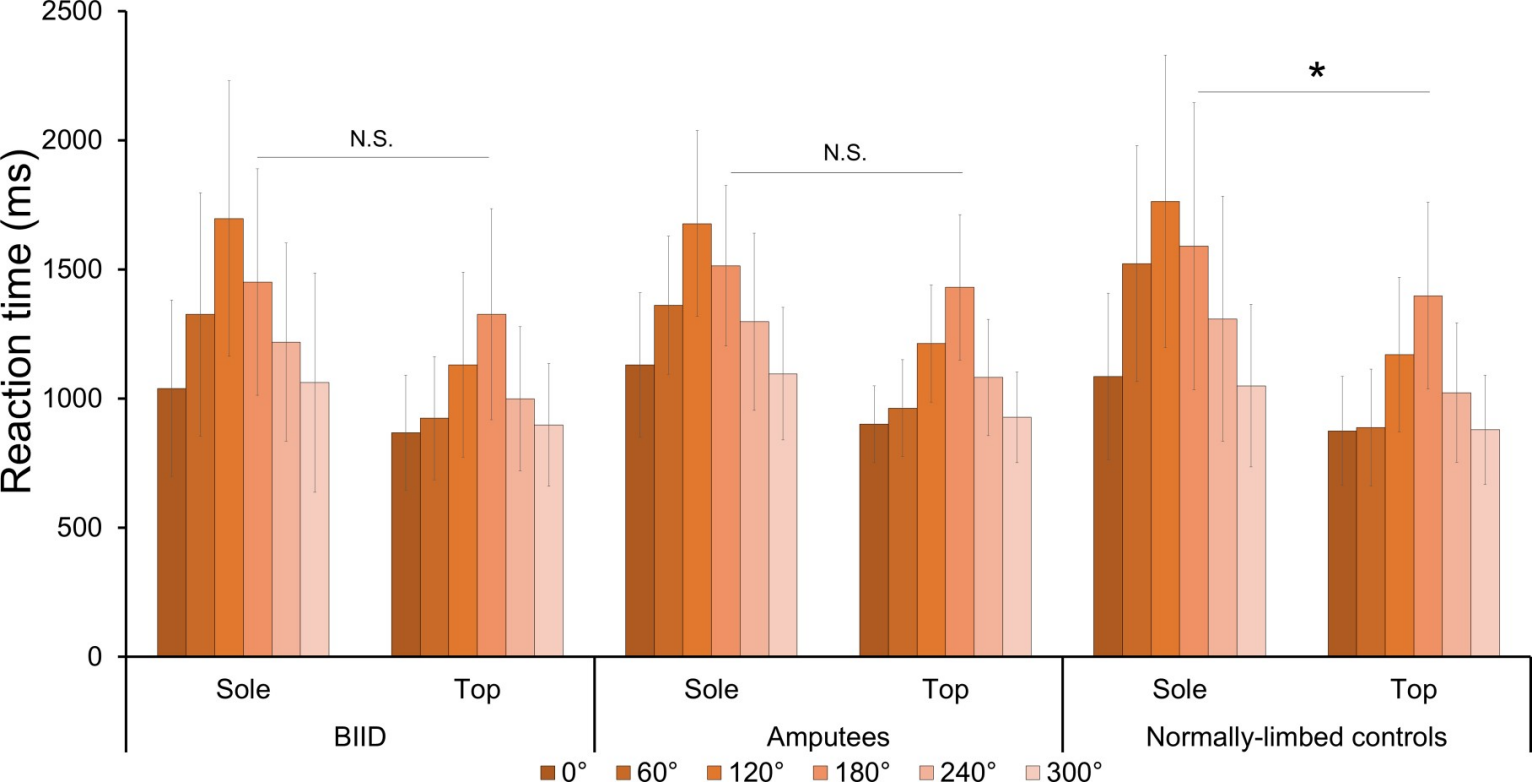
Reaction times for view and orientation per group. Bar graph displaying (untransformed) average reaction times in milliseconds to foot stimuli presented at different views (sole, top), orientations (0–300 degrees) and groups (BIID, amputees, and normally-limbed controls). Note that normally-limbed participants were significantly faster at making judgements about feet viewed from the top at 180 degrees (i.e. 3^rd^ person perspective, *p = 0.003), while this was not the case for BIID and amputee participants. Top views yielded significantly faster reaction times for all other orientations and all groups (not shown here for clarity sake, *p* < 0.001 for all comparisons, critical *p* following Bonferroni-correction was 0.008). N.S. = not significant.

There was no main effect of group (*F*(2, 65) = 0.2, *p =* 0.79, η^2^ *=* 0.007). However, there was an interaction between orientation, view, and group (*F*(10, 325) = 2.2, *p =* 0.01, η^2^ *=* 0.06). To further investigate differences between the levels of this interaction, we first visually explored the data by plotting it (see Fig 3 for graphical representation). Because the difference between top and sole views were not the same across orientations, we inspected the difference between the views within each level of orientation, separately for each group, using paired samples t-tests (critical Bonferroni-corrected *p* = 0.008). While normally-limbed participants were significantly faster at making judgements about feet presented from the top at 180 degrees than from the sole (*t*(32) = -3.1, *p* = 0.003 and between all other orientations (*t* ≥ 7.8, *p* < 0.001 for all), BIID (*t*(15) = -2.0, *p* = 0.06) and amputee (*t*(18) = -1.1, *p* = 0.26) participants showed no significant difference in reaction times for feet viewed from the top or sole but only when they were oriented at 180 degrees (i.e., third-person perspective). However, both groups were significantly faster at making judgements about feet viewed from the top versus the sole for all other orientations (*p* < 0.001 for all). Importantly, there was no interaction between laterality and group (*F*(2, 65) = 0.5, *p* = 0.5, η^2^ *=* 0.01), and no other interactions with side (*p* > 0.1 for all interactions). Taken together, these results show no support for our hypotheses that participants with BIID would be slower for making judgements about images corresponding to their affected foot compared to their non-affected foot and compared to the other two groups (see Fig 3).

#### Error rates

**Fig 4:**
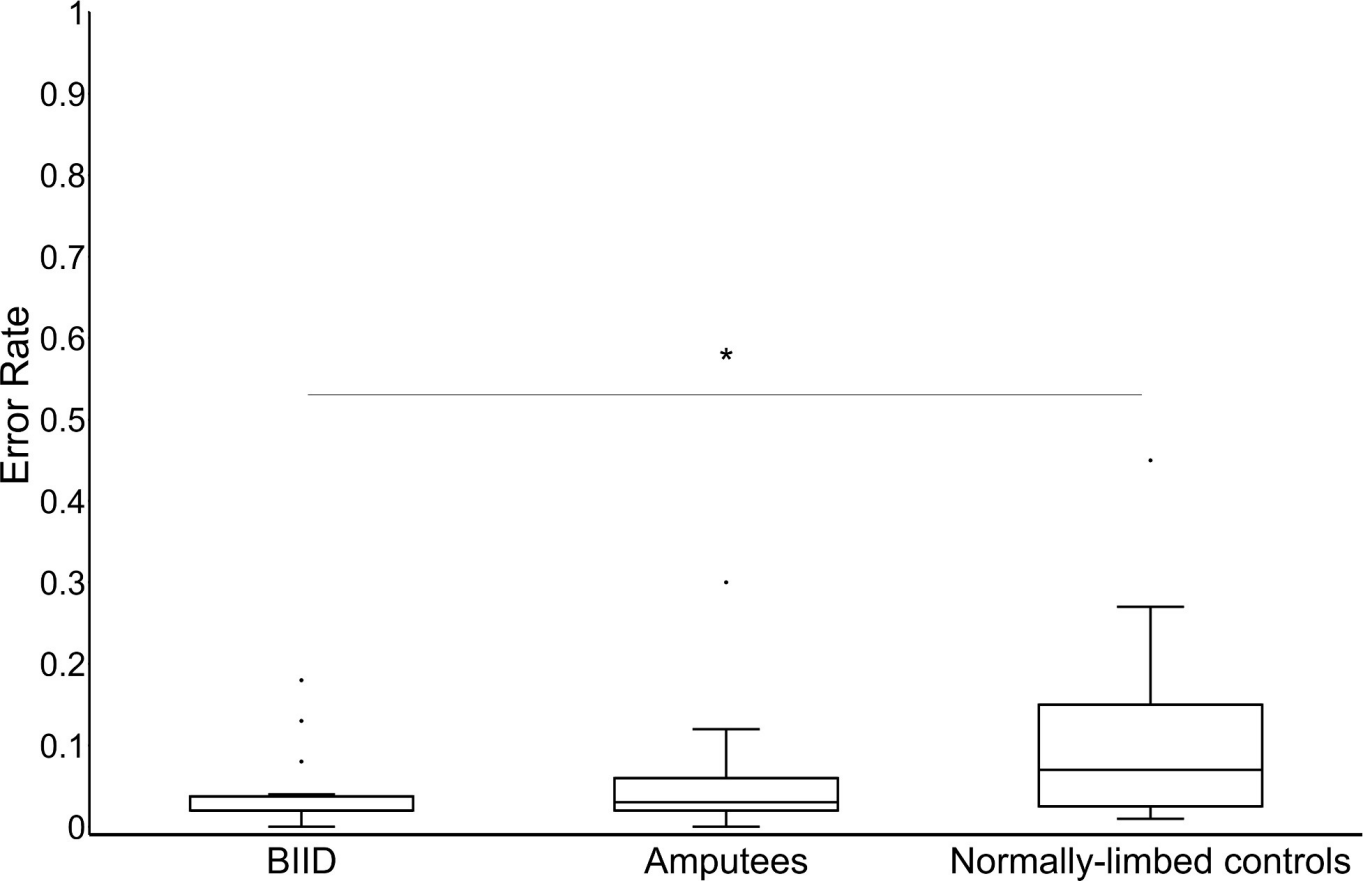
Error rates per group. Box plot displaying median error rate and interquartile range for all groups. Note that the normally-limbed group had a significantly higher error rate than BIID, but not amputee, participants. Medians for each group are represented as the center. Space within the box above the medians represent the third quartiles, and space within the box below the medians represent the first quartile. The whiskers represent maximum (above box) and minimum (below box) values. The outlined circles represent extreme scores. * *p* < 0.05.

Error data were not normally distributed for several conditions (i.e. error rates were 0, greatly skewing the distribution). Therefore, we decided to collapse across all conditions and look at error rates between groups using a Kruskal-Wallis test. Median error rates and interquartile ranges were as follows: BIID participants (0.02 ± 0.01–0.03), amputees (0.02 ± 0.02–0.05), normally-limbed controls (0.07 ± 0.02–0.14). The Kruskal-Wallis test was significant (χ2(2) = 7.1, *p* = 0.03). Follow-up Mann-Whitney U tests revealed that the difference in overall error rates between amputees and BIID participants (*U* = 100.5, *p* = 0.08) was not significant, nor was the difference between amputees and normally-limbed participants (*U* = 227.5, *p* = 0.1). However, there was a significant difference between BIID and normally-limbed participants (*U* = 156.5, *p* = 0.02), insofar that normally-limbed participants had slightly higher error rates than BIID participants (see Fig 4).

## Discussion

In the current study we investigated whether disrupted feelings of lower-limb integrity (as in BIID) influenced performance on a task involving mental rotation of feet. In an online study, participants were asked to judge the laterality of foot images presented from different views, orientations, laterality, and as different types. As the task usually involves mentally rotating one’s own body part to match the pictured foot, we expected individuals with BIID to be slower than lower-limb amputees and normally-limbed participants at making judgements about feet that corresponded to their affected side (i.e. the side desired to be amputated). Contrary to our expectation, we did not find any significant differences in reaction times between groups based on laterality. BIID participants performed similarly to lower-limb amputees and normally-limbed participants on this task, which suggests a preserved ability to mentally rotate affected body parts in this population.

BIID is a rare condition characterized by a strong and persistent desire to amputate or paralyze one or more healthy limbs, predominantly the legs [68]. Studies involving BIID participants have revealed an altered network in the brain for representing the body, especially the lower limbs. Specifically, there is evidence of a central under-representation of the legs (mainly in the paracentral lobule) and issues with multimodal integration of that limb, i.e. failed activation of the premotor and superior parietal lobule in response to tactile stimuli on the limbs [4,7,10,12]. These findings suggest that there might be issues integrating lower level sensory input with higher-order models of the body, models which are likely abridged in BIID. We aimed to tap into this compromised representation of the leg in BIID by asking participants to mentally rotate images of feet, a task that is presumably completed by temporarily using one’s own body part representation to make a laterality judgement. The finding that all participants were slower to make judgements about feet in awkward postures suggests that they were using an internal, egocentric model of the foot to make a judgement (e.g. [30,44,49]). BIID participants therefore seem to maintain their ability to mentally rotate the affected limb like normally-limbed individuals. Thus, while individuals with BIID may have an altered integration of higher-order somatosensory and proprioceptive information of the legs/feet [10], it is possible that visual representations of the legs/feet are still intact. Participants with BIID feel like their leg should be paralyzed or amputated, but do not necessarily report issues with the visual appearance of it. An examination of visual perception of the legs in BIID is currently underway in our lab, which might validate this query. Thus, participants may still be able to tap into a visual representation of the foot in order to solve the task (e.g. as recently suggested by the brain activity of lower-limb amputees [45]). Moreover, the motor network for action execution still functions normally in BIID [10]. Studies have shown that executing an action and imagining an action activate similar motor-related networks in the brain (e.g. [69]), similar to those involved in mental rotation [19,40,43]. Thus, it could be that mental spatial transformation of feet in BIID is spared by a normally-functioning motor system (though brain activity during imagined movements of the affected leg has yet to be tested). Taken together, the integration of visual and motor representations of the feet in BIID might facilitate performance for mental rotation of feet.

Several studies have investigated the role that peripheral disruptions, like an amputation or pain, play on mental rotations of body parts [34,44,47–49,51,70,71]. Amputation of an upper limb, particularly the dominant hand, seems to negatively influence performance on its mental rotation [34]. However, amputation of a lower limb seems to have a less robust detrimental effect on performance in such a task [44,45]. In the first study to investigate mental rotation of body parts in lower-limb amputees, Curtze and colleagues [44] revealed similar performance between lower-limb amputees and controls on a mental rotation task of the feet. However, they did find an interaction between side and group for reaction times. Because this was not confirmed for the error rate data, they do not discuss the interaction. It is possible that amputees may have been slower for their affected leg, but they made just as many errors as controls. We could not examine this in our study as error rates revealed a ceiling effect in most conditions. In addition, Curtze et al. showed that one individual with rotationplasty–a rare surgical condition in which a lower limb is amputated for medical reasons related to the knee and the intact foot is then rotated by 180 degrees and transplanted to the thigh so that the ankle can serve as knee substitute for a future prosthesis–had an intact ability to mentally rotate feet. These findings suggest that mental rotation abilities, at least for foot images, are preserved in the face of physical changes to the lower body. Our study is one of few to examine the role of a lower-limb amputation on mental rotation of feet. We replicate other findings insofar that lower-limb amputees performed similarly to normally-limbed controls on mentally rotating feet, even for their amputated side [44,45]. Importantly, Curtze et al. also found an interaction between side, orientation, and the absence/presence of phantom sensations (when included as a co-variate) during the task. However, this interaction was not elaborated upon in their report. In their study, only 2/18 participants experienced phantom sensations during the task. In our study, 8/19 participants stated in their questionnaire that they were experiencing a phantom limb sensation at the time of testing. As this was not the main aim of our study, in a supplementary analysis (see supporting S1 File), we reveal an interaction between foot type, view, and presence/absence of a phantom limb sensation. This suggests that those *not* experiencing phantom limb sensations were actually at a disadvantage (i.e. slower reaction times) for judging prosthetic feet (compared to real feet) viewed from the top. In line with Curtze et al.’s interpretation, this might suggest that participants were probably using motor imagery to solve the task. Lyu et al. [72] investigated the influence of phantom limbs on mental rotation of hands in upper limb amputees. They showed that those experiencing a phantom limb had prolonged reaction times to judging hand stimuli compared to those who did not and compared to controls (the latter two groups did not differ). However, their stimuli were judged from different orientations (0–300 degrees) and sides (left, right), but were of only one type (real hand) and view (from the top). Our interaction is specific to these factors. However, we only had few individuals (i.e. n = 8) that experienced phantom limb sensations at the time of the study, so more studies need to investigate this phenomenon during mental rotation. Specifically, as there is a lack of studies on lower-limb mental rotation in amputees, future studies could investigate the role of phantom experiences in upper- and lower-limb amputees for mental rotation of body parts, as there seems to be some evidence that this perception differentially affects reaction times.

Also noteworthy is that most (*n* = 17) of our amputees used prostheses, either on a daily basis (*n* = 15), almost daily (*n =* 1) or less than twice a week (*n =* 1). In addition, 14/19 reported wearing prostheses during the experiment. It is conceivable that the use of prostheses reactivates the representation of the missing lower limb, bridging any possible expected gap in performance between amputees and control participants for mental rotation of feet. Guo et al. [70] investigated mental rotation of hands in upper-limb amputees with a history of prosthesis use and those without. Those with a history of prosthesis use were equally fast (and showed similar brain activity) during the task as control participants. Non-prosthesis users, however, were slower and showed aberrant EEG activity during the task. Even three of their participants who no longer used prostheses still performed the same as controls on the task. These findings, together with ours, suggest that prostheses might indirectly uphold an internal representation of the amputated body part allowing for unaffected mental rotation. In BIID, it could be that the physical presence of the leg also contributes to maintenance of an internal motor schema that at least allows for mental rotation of that part. Moreover, since low-level peripheral input on the limb is seemingly normal in BIID (e.g. pain, temperature, vibration, position sense [64]), it is possible that peripheral input overrides the aberrant central representation (distributed across frontal, parietal, and insular areas) in this task, allowing for normal spatial transformation of body parts.

While we did not find support for our hypothesis of an interaction between group and side, we found an unexpected interaction between group, orientation, and view on reaction times. Specifically, we found that amputees and BIID participants showed no significant differences in reaction times for making judgements about feet displayed from a third-person perspective (i.e. 180 degrees) for the top and sole views. In contrast, normally-limbed participants were *not* equally fast at making judgments about feet displayed from a third-person perspective, insofar that they were significantly faster when the feet were viewed from the top than from the sole, in line with previous studies for top and bottom body part views [31–34,44]. All groups were faster for all other orientations at making judgements about the top versus the sole, however. The finding that amputees were not significantly faster at top versus sole views at 180 degrees might be reflective of a more flexible repertoire of possible postures for the feet, given that one leg is not physically present. This might place less constraints on possible mental transformations of the lower body. Future studies could try to elucidate this proposed mechanism.

We also found an unexpected interaction between foot type, orientation, and view. Specifically, participants were significantly faster at making judgements about real feet oriented at 180 degrees viewed from the top than from the sole but did not show a significant difference between responses for top versus sole views of prosthetic feet oriented at 180 degrees. This discrepancy in behaviour suggests that participants referred to their own foot’s representation to make the laterality judgements. In other words, when the prosthetic foot was oriented in a third person perspective (180 degrees), view (top or sole) did not affect performance. In line with this, one might better consider the hypothesis that people with BIID treat their affected limb as a non-bodily object. It has been shown that people typically show a less exaggerated effect of orientation for response times to different orientations of letters or non-bodily objects compared to body parts [35,36]. Including a condition with a non-bodily object (e.g. letters) might further clarify whether the presented body part is actually rotated by using an internal representation.

Finally, participants made very few errors during this task (median < 0.04, or 4%, across all participants). Therefore, we collapsed error rate data across all conditions to obtain a total error rate and compared this between groups. We found that normally-limbed participants had a significantly higher error rate than BIID participants, but no other statistically significant group differences emerged. However, while not statistically significant, it is worth noting the difference between amputees and BIID participants (*p* = 0.08), suggesting that BIID made fewer errors than amputees. In general, though, there were so few errors that we could not examine this difference more thoroughly, i.e. whether it was specific to one or two conditions. While our error rates might seem surprisingly low, our average error rate is in line with at least two other studies involving mental rotation of feet (e.g. [30]: ~5%, [71]: ~5.4%). Perhaps increasing task difficulty might reveal differences between groups, but the reaction time data, as it stands, cannot speak to this.

A central limitation of the current study is that participants completed the task online. We chose an online study approach to increase our sample size, given that BIID is such a rare and secretive condition [2]. Many BIID individuals communicate anonymously about their condition with others in online forums. Participation in an online experiment allows us to gain more understanding about the condition but also protects the anonymity of the participant. However, it is worth noting that the validity of the BIID group could have been affected by this. Specifically, we could not confirm a BIID diagnosis in these individuals. Telephone interviews administered by a trained healthcare professional, in which participants are asked specific questions regarding the history and course of their BIID (e.g. using the criteria from First and Fisher [1]) may have better validated our BIID sample. With respect to limitations regarding experimental compliance, our findings could be corroborated by testing the same protocol in the lab. However, the main effects of orientation and view align with other lab-based mental rotation task studies, validating the parameters of our task. Participants were instructed to sit normally with their feet flat on the floor prior to starting the experiment. However, we could not monitor or control their current posture, nor whether they wore their prosthesis. This might have influenced our results to some extent, but the influence of foot posture on mental rotation of feet has not been explored yet. Another possible limitation is the mother tongue of the participants. The experiment was conducted in English, but most participants were not native English speakers. While this may have affected responses on the questionnaires, we do not believe it affected performance on the mental rotation task (which simply required forced-choice key press). We would expect a much higher error rate in our task if participants did not understand the task due to language barriers. Finally, due to the unequal sample sizes per group, statistical power underlying these results may be reduced.

To conclude, we found that the ability to mentally rotate affected body parts, at least in terms of reactions times, in BIID is not statistically different from that of lower-limb amputee and normally-limbed participants. We replicate the findings of previous studies looking at mental rotation of feet in lower-limb amputees [44,45]. The role of lower phantom limb sensations in mental rotations, however, warrant future investigation. Few studies have behaviourally examined body representations in BIID. This is the first study, to our knowledge, to examine mental rotation of feet in BIID. Our findings contribute to the growing literature on BIID and suggest that mental rotation tasks might not tap into the incongruent bodily experience individuals with this condition report. Other implicit body representation tasks, like those related to proprioception and somatosensation, are currently being employed in our lab with individuals with BIID. Understanding the behavioural manifestation of this disturbed bodily experience might aid in the development of clinical tests for the diagnosis of BIID, as none have been developed yet.

## Supporting information

S1 Table Characteristics of participants. General characteristics and questionnaires scores for all participants.
(XLSX)

S1 File Covariate analyses. File containing results of within group ANCOVAs with years since amputation/years with BIID, prosthesis use, phantom sensations, and posture change during experiment as covariates.
(DOCX)

S1 Data File Experiment data file. Data file including log-transformed reaction times and error rates for the mental rotation task, in addition to questionnaire scores and general participant information.
(XLSX)

## References

[1] First MB, Fisher C. Body Integrity Identity Disorder: The persistent desire to acquire a physical disability. Psychopathology. 2012;45: 3–14. 10.1159/000330503 22123511

[2] Blom RM, Hennekam RC, Denys D. Body Integrity Identity Disorder. PLoS One. 2012;7: e34702 10.1371/journal.pone.0034702 22514657 PMC3326051

[3] Blom RM, Van Wingen GA, Van Der Wal SJ, Luigjes J, Van Dijk MT, Scholte HS, et al The desire for amputation or paralyzation: Evidence for structural brain anomalies in Body Integrity Identity Disorder (BIID). PLoS One. 2016;11: 1–13. 10.1371/journal.pone.0165789 27832097 PMC5104450

[4] Hilti LM, Hänggi J, Vitacco DA, Kraemer B, Palla A, Luechinger R, et al The desire for healthy limb amputation: Structural brain correlates and clinical features of xenomelia. Brain. 2013;136: 318–329. 10.1093/brain/aws316 23263196

[5] Oddo-Sommerfeld S, Hänggi J, Coletta L, Skoruppa S, Thiel A, Stirn A V. Brain activity elicited by viewing pictures of the own virtually amputated body predicts xenomelia. Neuropsychologia. Elsevier Ltd; 2018;108: 135–146. 10.1016/j.neuropsychologia.2017.11.025 29174728

[6] Macauda G, Bekrater-Bodmann R, Brugger P, Lenggenhager B. When less is more–Implicit preference for incomplete bodies in xenomelia. J Psychiatr Res. Elsevier Ltd; 2017;84: 249–255. 10.1016/j.jpsychires.2016.09.019 27776292

[7] Hänggi J, Vitacco DA, Hilti LM, Luechinger R, Kraemer B, Brugger P. Structural and functional hyperconnectivity within the sensorimotor system in xenomelia. Brain Behav. 2017;7: 1–17. 10.1002/brb3.657 28293484 PMC5346531

[8] Brugger P, Christen M, Jellestad L, Hänggi J. Limb amputation and other disability desires as a medical condition. The Lancet Psychiatry. 2016;3: 1176–1186. 10.1016/S2215-0366(16)30265-6 27889011

[9] Reed GM, First MB, Kogan CS, Hyman SE, Gureje O, Gaebel W, et al Innovations and changes in the ICD-11 classification of mental, behavioural and neurodevelopmental disorders. World Psychiatry. 2019;18: 3–19. 10.1002/wps.20611 30600616 PMC6313247

[10] van Dijk MT, van Wingen GA, van Lammeren A, Blom RM, de Kwaasteniet BP, Scholte HS, et al Neural basis of limb ownership in individuals with Body Integrity Identity Disorder. PLoS One. 2013;8: 1–6. 10.1371/journal.pone.0072212 23991064 PMC3749113

[11] Hänggi J, Bellwald D, Brugger P. Shape alterations of basal ganglia and thalamus in xenomelia. NeuroImage Clin. 2016;11: 760–769. 10.1016/j.nicl.2016.05.015 27330976 PMC4909827

[12] McGeoch PD, Brang D, Song T, Lee RR, Huang M, Ramachandran VS. Xenomelia: A new right parietal lobe syndrome. J Neurol Neurosurg Psychiatry. 2011;82: 1314–1319. 10.1136/jnnp-2011-300224 21693632

[13] Limanowski J, Blankenburg F. Integration of visual and proprioceptive limb position information in human posterior parietal, premotor, and extrastriate cortex. J Neurosci. 2016;36: 2582–2589. 10.1523/JNEUROSCI.3987-15.2016 26937000 PMC6604875

[14] Gentile G, Björnsdotter M, Petkova VI, Abdulkarim Z, Ehrsson HH. Patterns of neural activity in the human ventral premotor cortex reflect a whole-body multisensory percept. Neuroimage. Academic Press; 2015;109: 328–340. 10.1016/j.neuroimage.2015.01.008 25583608 PMC4349631

[15] Ehrsson HH. That’s my hand! Activity in premotor cortex reflects feeling of ownership of a limb. Science (80-). 2004;305: 875–877. 10.1126/science.1097011 15232072

[16] Gentile G, Guterstam A, Brozzoli C, Ehrsson HH. Disintegration of multisensory signals from the real hand reduces default limb self-attribution: an fMRI study. J Neurosci. 2013;33: 13350–66. 10.1523/JNEUROSCI.1363-13.2013 23946393 PMC3742923

[17] Kammers MPM, Verhagen L, Dijkerman HC, Hogendoorn H, De Vignemont F, Schutter DJLG. Is this hand for real? Attenuation of the rubber hand illusion by transcranial magnetic stimulation over the inferior parietal lobule. J Cogn Neurosci. MIT Press; 2009;21: 1311–1320. 10.1162/jocn.2009.21095 18752397

[18] Naito E, Morita T, Amemiya K. Body representations in the human brain revealed by kinesthetic illusions and their essential contributions to motor control and corporeal awareness. Neurosci Res. Elsevier Ireland Ltd and Japan Neuroscience Society; 2016;104: 16–30. 10.1016/j.neures.2015.10.013 26562333

[19] Bonda E, Petrides M, Frey S, Evans A. Neural correlates of mental transformations of the body-in-space. Proc Natl Acad Sci. 1995;92: 11180–11184. 10.1073/pnas.92.24.11180 7479961 PMC40595

[20] Critchley M. The parietal lobes. London: Edward Arnold; 1953.

[21] Craig AD. How do you feel—now? The anterior insula and human awareness. Nat Rev Neurosci. 2009;10: 59–70. 10.1038/nrn2555 19096369

[22] Sedda A, Bottini G. Apotemnophilia, body integrity identity disorder or xenomelia? psychiatric and neurologic etiologies face each other. Neuropsychiatr Dis Treat. 2014;10: 1255–1265. 10.2147/NDT.S53385 25045269 PMC4094630

[23] Berti A. This limb is mine but I do not want it: from anatomy to body ownership. Brain. 2013;136: 11–13. 10.1093/brain/aws346 23365090

[24] Vallar G, Ronchi R. Somatoparaphrenia: A body delusion. A review of the neuropsychological literature. Exp Brain Res. 2009;192: 533–551. 10.1007/s00221-008-1562-y 18813916

[25] Romano D, Sedda A, Brugger P, Bottini G. Body ownership: When feeling and knowing diverge. Conscious Cogn. Elsevier Inc.; 2015;34: 140–148. 10.1016/j.concog.2015.04.008 25955181

[26] Romano D, Gandola M, Bottini G, Maravita A. Arousal responses to noxious stimuli in somatoparaphrenia and anosognosia: Clues to body awareness. Brain. 2014;137: 1213–1223. 10.1093/brain/awu009 24531623

[27] Lenggenhager B, Hilti LM, Brugger P. Disturbed body integrity and the “rubber foot illusion.” Neuropsychology. 2015;29: 205–211. 10.1037/neu0000143 25265068

[28] Smit M, van Stralen HE, Van den Munckhof B, Snijders TJ, Dijkerman HC. The man who lost his body: Suboptimal multisensory integration yields body awareness problems after a right temporoparietal brain tumour. J Neuropsychol. 2018; 1–10. 10.1111/jnp.12104 29532598 PMC6767520

[29] van Stralen HE, van Zandvoort MJE, Kappelle LJ, Dijkerman HC. The Rubber Hand Illusion in a patient with hand disownership. Perception. 2013;42: 991–993. 10.1068/p7583 24386718

[30] Parsons LM. Imagined spatial transformations of one’s hands and feet. Cogn Psychol. 1987;19: 178–241. 10.1016/0010-0285(87)90011-9 3581757

[31] Ionta S, Blanke O. Differential influence of hands posture on mental rotation of hands and feet in left and right handers. Exp Brain Res. 2009;195: 207–217. 10.1007/s00221-009-1770-0 19326106

[32] Ionta S, Fourkas AD, Fiorio M, Aglioti SM. The influence of hands posture on mental rotation of hands and feet. Exp Brain Res. 2007;183: 1–7. 10.1007/s00221-007-1020-2 17643238

[33] de Lange FP, Helmich RC, Toni I. Posture influences motor imagery: An fMRI study. Neuroimage. 2006;33: 609–617. 10.1016/j.neuroimage.2006.07.017 16959501

[34] Nico D, Daprati E, Rigal Ë, Parsons L, Sirigu A, Lucia FS, et al Left and right hand recognition in upper limb amputees. Brain. 2004;127: 120–132. 10.1093/brain/awh006 14607796

[35] Krüger M, Amorim MA, Ebersbach M. Mental rotation and the motor system: Embodiment head over heels. Acta Psychol (Amst). Elsevier B.V.; 2014;145: 104–110. 10.1016/j.actpsy.2013.11.004 24333809

[36] Dalecki M, Dern S, Steinberg F. Mental rotation of a letter, hand and complex scene in microgravity. Neurosci Lett. Elsevier Ireland Ltd; 2013;533: 55–59. 10.1016/j.neulet.2012.11.002 23147120

[37] Ganis G, Keenan JP, Kosslyn SM, Pascual-Leone A. Transcranial magnetic stimulation of primary motor cortex affects mental rotation. Cereb Cortex. 2000;10: 175–180. 10.1093/cercor/10.2.175 10667985

[38] Berneiser J, Jahn G, Grothe M, Lotze M. From visual to motor strategies: Training in mental rotation of hands. Neuroimage. Elsevier Ltd; 2018;167: 247–255. 10.1016/j.neuroimage.2016.06.014 27321046

[39] Wraga M, Thompson WL, Alpert NM, Kosslyn SM. Implicit transfer of motor strategies in mental rotation. Brain Cogn. 2003;52: 135–143. 10.1016/S0278-2626(03)00033-2 12821095

[40] Parsons LM, Fox PT, Downs JH, Glass T, Hirsch TB, Martin CC, et al Use of implicit motor imagery for visual shape discrimination as revealed by PET. Nature. 1995 pp. 54–58. 10.1038/375054a0 7723842

[41] Vingerhoets G, Santens P, Van Laere K, Lahorte P, Dierckx RA, De Reuck J. Regional brain activity during different paradigms of mental rotation in healthy volunteers: A positron emission tomography study. Neuroimage. 2001;13: 381–391. 10.1006/nimg.2000.0690 11162278

[42] Tomasino B, Gremese M. Effects of stimulus type and strategy on mental rotation network: An activation likelihood estimation meta-analysis. Front Hum Neurosci. 2015;9: 693 10.3389/fnhum.2015.00693 26779003 PMC4704562

[43] Kosslyn SM, DiGirolamo GJ, Thompson WL, Alpert NM. Mental rotation of objects versus hands: Neural mechanisms revealed by positron emission tomography. Psychophysiology. 1998;35: 151–161. 10.1017/S0048577298001516 9529941

[44] Curtze C, Otten B, Postema K. Effects of lower limb amputation on the mental rotation of feet. Exp Brain Res. 2010;201: 527–534. 10.1007/s00221-009-2067-z 19902193 PMC2832871

[45] Boccia M, Vita A Di, Palermo L, Nemmi F, Traballesi M, Brunelli S, et al Neural modifications in lower limb amputation: an fMRI study on action and non-action oriented body representations. Brain Imaging Behav. Brain Imaging and Behavior; 2019; 10.1007/s11682-019-00142-3 31214871

[46] Ionta S, Villiger M, Jutzeler CR, Freund P, Curt A, Gassert R. Spinal cord injury affects the interplay between visual and sensorimotor representations of the body. Sci Rep. 2016;6: 1–10. 10.1038/s41598-016-0001-8 26842303 PMC4740737

[47] Johnson SH, Sprehn G, Saykin AJ. Intact motor imagery in chronic upper limb hemiplegics: Evidence for activity-independent action representations. J Cogn Neurosci. 2002;14: 841–852. 10.1162/089892902760191072 12191452

[48] Johnson SH. Imagining the impossible: Intact motor representations in hemiplegics. Neuroreport. 2000;11: 729–732. 10.1097/00001756-200003200-00015 10757509

[49] Coslett HB, Medina J, Kliot D, Burkey A. Mental motor imagery and chronic pain: The foot laterality task. J Int Neuropsychol Soc. Universiteitsbibliotheek Utrecht; 2010;16: 603–612. 10.1017/S1355617710000299 20380787

[50] Reinersmann A, Haarmeyer GS, Blankenburg M, Frettlöh J, Krumova EK, Ocklenburg S, et al Left is where the L is right. Significantly delayed reaction time in limb laterality recognition in both CRPS and phantom limb pain patients. Neurosci Lett. Elsevier Ireland Ltd; 2010;486: 240–245. 10.1016/j.neulet.2010.09.062 20887773

[51] Fiorio M, Tinazzi M, Ionta S, Fiaschi A, Moretto G, Edwards MJ, et al Mental rotation of body parts and non-corporeal objects in patients with idiopathic cervical dystonia. Neuropsychologia. 2007;45: 2346–2354. 10.1016/j.neuropsychologia.2007.02.005 17412373

[52] Katschnig P, Edwards MJ, Schwingenschuh P, Aguirregomozcorta M, Kägi G, Rothwell JC, et al Mental rotation of body parts and sensory temporal discrimination in fixed dystonia. Mov Disord. 2010;25: 1061–7. 10.1002/mds.23047 20310052

[53] Hoyek N, Di Rienzo F, Collet C, Creveaux T, Guillot A. Hand mental rotation is not systematically altered by actual body position: Laterality judgment versus same-different comparison tasks. Attention, Perception, Psychophys. 2014;76: 519–526. 10.3758/s13414-013-0577-z 24170381

[54] van Stralen HE, Dijkerman HC, Biesbroek JM, Kuijf HJ, van Gemert HMA, Sluiter D, et al Body representation disorders predict left right orientation impairments after stroke: A voxel-based lesion symptom mapping study. Cortex. Elsevier Ltd; 2017;104: 140–153. 10.1016/j.cortex.2017.05.025 28732749

[55] Shenton JT, Schwoebel J, Coslett HB. Mental motor imagery and the body schema: Evidence for proprioceptive dominance. Neurosci Lett. 2004;370: 19–24. 10.1016/j.neulet.2004.07.053 15489010

[56] Bekrater-Bodmann R, Schredl M, Diers M, Reinhard I, Foell J, Trojan J, et al Post-amputation pain is associated with the recall of an impaired body representation in dreams-results from a nation-wide survey on limb amputees. PLoS One. Public Library of Science; 2015;10: e0119552 10.1371/journal.pone.0119552 25742626 PMC4350998

[57] Sheehan D V., Lecrubier Y, Sheehan KH, Amorim P, Janavs J, Weiller E, et al The Mini-International Neuropsychiatric Interview (M.I.N.I.): The development and validation of a structured diagnostic psychiatric interview for DSM-IV and ICD-10. J Clin Psychiatry. 1998;59: 22–33. 10.1016/S0924-9338(99)80239-9 9881538

[58] Spotts JL. Utility of the Modified Mini Screen (MMS) for screening mental health disorders in a prison population. Dissertation Abstracts International, B: Sciences and Engineering. 2008.

[59] Bohus M, Kleindienst N, Limberger MF, Stieglitz RD, Domsalla M, Chapman AL, et al The short version of the Borderline Symptom List (BSL-23): Development and initial data on psychometric properties. Psychopathology. 2009;42: 32–39. 10.1159/000173701 19023232

[60] Lu WH, Wang PW, Ko CH, Hsiao RC, Liu TL, Yen CF. Differences in mental health among young adults with borderline personality symptoms of various severities. J Formos Med Assoc. Elsevier Ltd; 2018;117: 332–338. 10.1016/j.jfma.2017.04.020 28511866

[61] Leon AC, Olfson M, Portera L, Farber L, Sheehan DV. Assessing psychiatric impairment in primary care with the Sheehan Disability Scale. Int J Psychiatry Med. 1997;27: 93–105. 10.2190/T8EM-C8YH-373N-1UWD 9565717

[62] Ferri F, Frassinetti F, Ardizzi M, Costantini M, Gallese V. A sensorimotor network for the bodily self. J Cogn Neurosci. 2012;24: 1584–1595. 10.1162/jocn_a_00230 22452562

[63] Elias LJ, Bryden MP, Bulman-Fleming MB. Footedness is a better predictor than is handedness of emotional lateralization. Neuropsychologia. 1998;36: 37–43. 10.1016/S0028-3932(97)00107-3 9533385

[64] Aoyama A, Krummenacher P, Palla A, Hilti LM, Brugger P. Impaired spatial-temporal integration of touch in Xenomelia (Body Integrity Identity Disorder). Spat Cogn Comput. 2012;12: 96–110. 10.1080/13875868.2011.603773

[65] Nummenmaa L, Glerean E, Hari R, Hietanen JK. Bodily maps of emotions. Proc Natl Acad Sci U S A. 2014;111: 646–51. 10.1073/pnas.1321664111 24379370 PMC3896150

[66] Ionta S, Perruchoud D, Draganski B, Blanke O. Body context and posture affect mental imagery of hands. PLoS One. 2012;7 10.1371/journal.pone.0034382 22479618 PMC3316677

[67] Wohlschläger A, Wohlschläger A. Mental and Manual Rotation. J Exp Psychol Hum Percept Perform. 1998;24: 397–412. 10.1037/0096-1523.24.2.397 9606108

[68] Sedda A. Body integrity identity disorder: From a psychological to a neurological syndrome. Neuropsychol Rev. 2011;21: 334–336. 10.1007/s11065-011-9186-6 22071988

[69] Munzert J, Lorey B, Zentgraf K. Cognitive motor processes: The role of motor imagery in the study of motor representations. Brain Res Rev. 2009;60: 306–326. 10.1016/j.brainresrev.2008.12.024 19167426

[70] Guo X, Lin Z, Lyu Y, Bekrater-Bodmann R, Flor H, Tong S. The Effect of Prosthesis Use on Hand Mental Rotation after Unilateral Upper-Limb Amputation. IEEE Trans Neural Syst Rehabil Eng. 2017;25: 2046–2053. 10.1109/TNSRE.2017.2702117 28489541

[71] Uritani D, Nishida T, Sakaguchi N, Kawakami T, Jones L, T. K. Difference in response to a motor imagery task: A comparison between individuals with and without painful temporomandibular disorders. Pain Res Manag. 2018;2018: 1–8. 10.1155/2018/6810412 PMC6091325 30154945

[72] Lyu Y, Guo X, Bekrater-Bodmann R, Flor H, Tong S. Phantom limb perception interferes with motor imagery after unilateral upper-limb amputation. Sci Rep. Nature Publishing Group; 2016;6: 2100 10.1038/srep21100 26879749 PMC4754632

