# Supplementary material for: Mental rotation of feet in individuals with Body Integrity Identity Disorder, lower-limb amputees, and normally-limbed controls
Source: PLoS One. 2019 Aug 16;14(8):e0221105. doi: 10.1371/journal.pone.0221105 (PMC6697338; doi:10.1371/journal.pone.0221105)
Supplement: S1 File — File containing results of within group ANCOVAs with years since amputation/years with BIID, prosthesis use, phantom sensations, and posture change during experiment as covariates. (DOCX) [file pone.0221105.s002.docx]

**S1 Supporting Information File**

**Mental rotation of feet in individuals with Body Integrity Identity Disorder, lower-limb amputees, and normally-limbed controls**

K.D. Stone, H.C. Dijkerman, R. Bekrater-Bodmann, A. Keizer

**Supplementary Material**

We conducted a covariance analysis within each group to explore how factors like time since amputation/with BIID, presence of a phantom limb, prosthetic use, and posture change might influence reaction times. We ran separate 6x2x2x2 repeated measures ANCOVAs per group on the mean log-transformed reaction times with the covariates listed below.

1. **Years since amputation/years with BIID**

**Amputee participants.** Data were missing from 1 participant for this covariate. In line with Curtze et al.(1), years since amputation did not interact with any of the factors (all *F* < 1.9 , *p* > 0.09, η² > 0.1). There was no main effect of years since amputation (*F*(1,16) = 2.2, *p* = 0.1, η² = 0.1).

**BIID participants.** Data were missing from 2 participants for this covariate. Years with BIID did not interact with any of the factors (all *F* < 1.3, *p* > 0.2, η² > 0.1). There was no main effect of years with BIID (*F*(1,12) =0.1 *p* = 0.7, η² = 0.01).

1. **Wearing protheses during the experiment**

**Amputee participants.** 14/19 participants were wearing prostheses during the experiment. Wearing protheses did not interact with any of the factors (all *F* < 1.9, *p* > 0.1, η² < 0.1). There was no main effect of wearing prostheses (*F*(1,17) = 1.4, *p* = 0.2, η² = 0.08).

1. **Experiencing phantom limb sensations during the experiment (amputees only)**

In the general questionnaire for amputees, participants were asked "Are you experiencing phantom limb sensation(s) right now?" (yes/no), followed by “If yes, can you describe the sensations you are experiencing right now?”.

Eight of the nineteen participant stated that they were experiencing phantom limb sensations at the timing of testing. The descriptions (edited for grammar and language translation) were as follows:

- I can feel almost every detail of my amputated leg.
- It feels like the foot is fixed in a very tight ski boots or a vice. It is not hurting, but it has a strong feeling.
- Slight tingling inside the foot
- If I think about it at the moment, I feel [the phantom limb].
- [The phantom limb] feels shortened.
- The tingling is always there, pain peaks come more often a day.
- I feel my lower leg and knee as if they were still there.
- Pins and needles in the [phantom] leg.

There was no main effect of the presence/absence of phantom limb sensations during the experiment (*F*(1,17) = 0.1, *p* = 0.7, η² = 0.007). There was an interaction between type, view, and presence/absence of phantom limb sensations (*F*(1,17)) = 5.4, *p* = 0.03, η^2^ = 0.2). Follow-up paired samples t-test revealed that participants not experiencing phantom limb sensations were slower at judging prosthetic feet viewed from the top, though this did not survive Bonferroni corrections (*t*(10) = 2.2, *p* = 0.045). In contrast, those experiencing phantom limb sensations showed no difference between prosthetic and real feet viewed from the top (*t*(7) = -0.4, *p* = 0.6). No other interactions with phantom presence were significant (*p* > 0.2).

1. **Posture change**

Participants were instructed to sit normally with their feet flat on the floor. After the experiment, they were asked if they changed posture during the task.

**Normally-limbed participants.** Eight participants indicated that they changed posture during the experiment. Posture change did not interact with any of the factors (all *F* < 3.9, *p* > 0.06, η² < 0.1). There was no main effect of posture change (*F*(1,31) = 0.9, *p* = 0.7, η² = 0.003).

**BIID participants.** Two participants indicated that they changed posture during the experiment. Posture change did not interact with any of the factors (all *F* < 1.9, *p* > 0.1, η² < 0.1). There was no main effect of posture change (*F*(1,14) = 0.1, *p* = 0.7, η² = 0.008).

**Amputee participants.** Two participants indicated that they changed posture during the experiment. Posture change did not interact with any of the factors (all *F* < 2.6 *p* > 0.1, η² < 0.1). There was no main effect of posture change (*F*(1,17) = 0.4, *p* = 0.5, η² = 0.02).

**References**

1. Curtze C, Otten B, Postema K. Effects of lower limb amputation on the mental rotation of feet. Exp Brain Res. 2010;201(3):527–34.
